# Evaluation of Aqueous Flare Intensity in Eyes Undergoing Intravitreal Bevacizumab Therapy to Treat Neovascular Age-Related Macular Degeneration

**DOI:** 10.3389/fphar.2021.656774

**Published:** 2021-04-30

**Authors:** Joanna Dolar-Szczasny, Claudio Bucolo, Sandrine Zweifel, Adriano Carnevali, Robert Rejdak, Wojciech Załuska, Aleksandra Czarnek-Chudzik, Mario Damiano Toro

**Affiliations:** ^1^ Department of General Ophthalmology, Medical University of Lublin, Lublin, Poland; ^2^ Section of Pharmacology, Department of Biomedical and Biotechnological Sciences, University of Catania, Catania, Italy; ^3^ Department of Ophthalmology, University of Zurich, Zurich, Switzerland; ^4^ Department of Ophthalmology, University “Magna Graecia”, Catanzaro, Italy; ^5^ Department of Nephrology, Medical University of Lublin, Lublin, Poland; ^6^ Department of Diagnostics and Microsurgery of Glaucoma, Medical University of Lublin, Lublin, Poland; ^7^ Faculty of Medical Sciences, Collegium Medicum, Cardinal Stefan Wyszyński University, Warsaw, Poland

**Keywords:** bevacizumab, neovascular age-related macular degeneration, laser-flare photometry, intraocular inflammation, blood-aqueous barrier integrity

## Abstract

**Purpose:** To evaluate the effect of repeated intravitreal bevacizumab injections on blood-aqueous barrier permeability in eyes with neovascular age-related macular degeneration (AMD).

**Patients and Methods:** Forty-eight consecutive patients with neovascular AMD received 3 intravitreal bevacizumab injections (1 mg) every 30–40 days. Subjects were followed for a period of 4 months and were examined at baseline, 1 day and 1 month after each injection. A control group comprised of 19 neovascular AMD patients waiting to begin anti-vascular endothelial growth factor (VEGF) therapy. Anterior chamber (AC) inflammation was evaluated with biomicroscopy and laser flare photometry.

**Results:** None of the subjects treated with bevacizumab had detectable ocular inflammation during follow-up. An analysis for variance (ANOVA) of the mixed-effects model has shown neither an effect between treatment and control group (*p* = 0.921), nor over the time course of the follow-up (*p* = 0.773). Before treatment, median AC inflammation was 6.7 photons/ms (range: 3.5–18.2 photons/ms). One month after the first, second, and third injections, median laser flare was 6.4, 6.8, and 6.6 photons/ms, respectively, none of which were significantly different from baseline (all *p* > 0.05). Blood-aqueous barrier permeability did not change between injections and was not different from the control group.

**Conclusion:** Inflammation induced by intravitreal bevacizumab was not detected by examination or flare photometry. This suggests that monthly bevacizumab dosing seems to be safe. The absence of AC inflammation could also reflect the known anti-inflammatory properties of anti-VEGF agents.

## Introduction

Vascular endothelial growth factor (VEGF) has become the main target for treating neovascular age-related macular degeneration (AMD) in recent years (Plyukhova et al., 2020). As a result, intravitreal injections of anti-VEGF agents are now widely used to halt neovascular AMD progression and, hopefully, improve central visual acuity. Unfortunately, each intraocular injection, even when performed under sterile conditions, carries a risk of vision-threatening complications, including intraocular inflammation, endophthalmitis, intraocular pressure elevation, vitreous hemorrhage, and retinal detachment (Falavarjani and Nguyen, 2013). The most serious of these complications is sterile or infectious endophthalmitis, which can lead to significant visual loss (Dossarps et al., 2015).

Among anti-VEGF agents, bevacizumab is often used intravitreally to handle several retinal diseases (Falavarjani and Nguyen, 2013; Reibaldi et al., 2014; Dossarps et al., 2015; Platania et al., 2015; Plyukhova et al., 2020; Yousef et al., 2020; Toro et al., 2021). Bevacizumab is used in ophthalmology in an off-label fashion and, thus, remains a somewhat controversial treatment option. Because the approved indications by drug regulatory agencies did not include ocular diseases, a concern regarding the safety profile and risk for emerged considering the potential ocular inflammatory response following intravitreal administration. Safety issue using chronic intravitreal bevacizumab dosing regimen has also arisen because most AMD patients require at least three injections during the first year of treatment (Plyukhova et al., 2020). On this regards it could be useful develop a biodegradable deliver system to inject bevacizumab, avoiding a multiple treatment (Conti et al., 1997).

Secondly, with regard to recent reports on more frequent inflammatory reactions after the use of the newly registered anti-VEGF drug-brolucizumab (Baumal et al., 2020), the issue of side effects has become even more topical. At the beginning of 2020, the American Society of Retinal Specialists (ASRS) alerted ophthalmologists to reported cases of ocular inflammation after brolucizumab injections (Beovu Update for ASRS Members, 2020).

A potent ocular inflammatory response is easily visible by slit-lamp examination after intraocular injection. This is especially true of changes in anterior chamber fluid clarity, but slight fluid changes can be overlooked (Falavarjani and Nguyen, 2013). However, a laser flare photometer can detect even subtle blood-ocular barrier changes that may not be detectable with standard ophthalmological clinical examination (Tugal-Tutkun and Herbort, 2010). Laser flare photometry (LFP) is a non-invasive tool and allows anterior chamber flare (from disruption of the blood-ocular barriers) to be objectively, accurately, and reproducibly quantified. Therefore, LFP allows ocular inflammatory responses induced by medications or surgical procedures to be examined and compared (Ladas et al., 2005; Tugal-Tutkun and Herbort, 2010; Orès et al., 2020).

Here, we use LFP to examine the effect of multiple intravitreal bevacizumab injections on ocular inflammation in neovascular AMD patients.

## Patients and Methods

The study protocol was reviewed and approved by the Institutional Review Board (IRB) at Medical University, Lublin, Poland (n° KE-0254/208/2013) on July 11th, 2013. Being the LFP a noninvasive diagnostic tool and according the ongoing regulation, the IRB waived the requirement of informed consent. All study conduct adhered to the tenets of the Declaration of Helsinki.

### Study Subjects

Consecutive patients diagnosed with neovascular AMD in the Lublin University Department of General Ophthalmology between January and September 2015 were retrospectively considered for inclusion in this cross-sectional analysis. All subjects had AMD with active macular neovascularization (MNV) confirmed with fluorescein angiography (FA), indocyanine green angiography, and optical coherence tomography (OCT). Only patients who required at least three intravitreal bevacizumab injections on a pro re nata treatment regimen and who had received a LFP monitoring during the loading phase, were included. Patients with advanced cataract, a history of uveitis or inflammation, vitreous hemorrhage, neovascular glaucoma, corneal opacities, recent ocular surgery (within 3 months), or prior anti-VEGF injections were excluded.

A control group of active neovascular AMD patients who were waiting to begin anti-VEGF therapy were also included and observed over a 4 months period. The delayed therapy in this group of patients was related to insufficient health availability in our clinic. These patients were informed about alternative medical centers and about the risks of delayed treatment but, due to transportation barriers and reimbursement issues, decided to wait for therapy in our hospital.

### Intravitreal Bevacizumab Injections

The use of intravitreal bevacizumab (Avastin, Genentech, Inc., South San Francisco, CA) as an off-label treatment was approved by the local ethics committee and it is part of clinical routine care in our department. All injections were performed under sterile conditions in our operating room after the patient had signed an informed consent for off-label drug administration. All treatments in this study were carried out as part of routine clinical care for neovascular AMD. After the eye was topically anesthetized with proxymetacaine (0.5%, Alcon-Couvrer nv, Puurs, Belgium), it was disinfected with several drops of 5% povidone iodine (Betadine Ophthalm., Alcon Laboratiries Inc.) placed in the conjunctival sac. Next, a 1.25 mg bevacizumab dose in 0.05 ml was injected into the vitreous cavity. The injected bevacizumab was obtained from a 4 ml vial that contained a 25 mg bevacizumab/ml solution. Sterile tuberculin syringes were used under sterile conditions to generate 0.1 ml (2.5 mg bevacizumab) aliquots of the drug just before intravitreal injections were prepared. The dosages were prepared by the doctor performing whole procedure in the surgical theatre. One day before and 5 days after injection, patients prophylactically used a topical antibiotic. Multiple injections were carried out with a pro renata regimen based on the clinical activity of the disease and the availability of the drug.

### Clinical Examinations

All patients were carefully and prospectively examined before, one day and one month after each of the three intravitreal bevacizumab injections during the loading phase. Treated and control subjects were monitored for a range of 4 months.

Ocular inflammation was qualitatively and quantitatively assessed by slit-lamp and fundoscopic examinations and a laser flare photometer, respectively (Kowa FM-500, Kowa Company, Ltd., Tokyo, Japan). All evaluations were made following pupil dilation with topical 1% tropicamide (Polfa-Warsaw SA, Poland). The final flare photometry value was automatically calculated by averaging 5 individual measurements. A total of 7 measurements were obtained, but the highest and lowest measurement values were excluded by the flare meter. All measurements were performed in a darkened room after calibrating the flare meter.

### Data Analyses

Data are presented as mean, median, standard deviation (SD), minimum value (min) and maximum value (max). Differences between groups were tested for statistical significance using the Mann-Whitney *U* test. For repeated measure data, a linear mixed-effects model was carried out to estimate differences between study groups and different time points. The two factors were adjusted for the baseline value. An analysis of variance (ANOVA) test of the mixed-effects model and the comparison to baseline for the treatment group, are presented as *p*-values. Statistical significance was defined as *p* < 0.05. Statistical analyses were performed using STATISTICA 12 statistical software (StatSoft Polska, Krakow, Poland) and the statistical software R version 4.0.

## Results

A total of 48 eyes of 48 patients (20 men, 28 women) were ultimately included in the injection group. Median patient age was 70 years (range: 47–87 years). A total of 19 eyes of 19 patients (8 men, 11 women) were ultimately included in the control group. Median control patient age was 65 years (range: 49–86 years).

None of the 48 patients in the injection group had clinically detectable anterior chamber inflammation during the follow-up period. Before treatment, median LFP measured 6.7 photons/ms (range: 3.5–18.2 photons/ms). This value was not significantly different from baseline one day following the first (median: 6.8 photons/ms, *p* = 0.738), second (7.1 photons/ms, *p* = 0.350), or third (6.5 photons/ms, *p* = 0.882) injection. The same was also true one month following the first (6.4 photons/ms, *p* = 0.419), second (6.8 photons/ms, *p* = 0.842), and third (6.6 photons/ms, *p* = 0.333) injections (Table 1). An ANOVA test of the linear mixed-effects model was carried out with neither a significant difference for treatment-control groups (*p* = 0.921) nor for the time points (*p* = 0.773). Mean values of anterior chamber flare were not significantly different between treated women and treated men at any follow-up time point examined (Table 2). Furthermore, there were no significant differences in blood-aqueous barrier permeability between treated (injection group) and untreated (control group) subjects at any time point examined (Table 3).

**TABLE 1:** Anterior chamber flare before and 1 day and 1 month after each intravitreal bevacizumab injection.

	n° (eyes)	Anterior chamber flare (photons/ms)					*p*	
		Min	Max	Mean	SD	Median	
Before treatment	48	3.5	18.2	7.51	3.42	6.70	
1 day after 1st injection	47	2.9	16.1	7.34	3.18	6.80	0.738
30 days after 1st injection	46	1.0	17.0	7.07	3.26	6.40	0.419
1 day after 2nd injection	47	3.2	16.9	8.03	3.07	7.10	0.419
30 days after 2nd injection	41	2.6	27.1	7.74	4.24	6.80	0.842
1 day after 3rd injection	38	2.7	22.5	7.60	3.86	6.50	0.882
30 days after 3rd injection	48	3.1	17.2	6.98	2.62	6.60	0.333

Min, minimum value; max, maximum value; SD, standard deviation; p, *p* value: *p*-values estimated with linear mixed-effects model.

**TABLE 2:** Anterior chamber flare before and 1 day and 1 month after each intravitreal bevacizumab injection in group of women and men.

	Women			Men			*p*
	n°	Mean	SD	n	Median	SD	
Age	28	67.71	11.25	20	69.55	7.94	0.565
Before treatment	28	7.34	3.55	20	7.75	3.31	0.579
1 day after 1st injection	27	7.21	3.51	20	7.52	2.75	0.383
30 days after 1st injection	27	7.01	3.18	19	7.15	3.46	0.647
1 day after 2nd injection	27	8.26	3.04	20	7.72	3.16	0.583
30 days after 2nd injection	25	7.47	3.20	16	8.16	5.59	0.936
1 day after 3rd injection	24	7.41	3.30	14	7.93	4.79	0.987
30 days after 3rd injection	28	6.73	2.78	20	7.33	2.42	0.310

SD, standard deviation; p, *p* value: *p*-values estimated with Mann-Whitney U test.

**TABLE 3:** Anterior chamber inflammation in the control group and in eyes treated with intravitreal bevacizumab.

	Laser flare photometry (photons/ms)						*p*
	Control group			Bevacizumab group			
	n° (eyes)	Mean	SD	n° (eyes)	Mean	SD	
Before treatment	17	7.32	3.22	48	7.51	3.42	0.840
1 day after 1st injection	18	8.56	3.75	47	7.34	3.18	0.253
30 days after 1st injection	18	7.03	3.39	46	7.07	3.26	0.893
1 day after 2nd injection	19	7.80	1.98	47	8.03	3.07	0.860
30 days after 2nd injection	19	6.68	2.47	41	7.74	4.24	0.546
1 day after 3rd injection	17	6.49	3.24	38	7.60	3.86	0.233
30 days after 3rd injection	18	7.49	4.76	48	6.98	2.62	0.565

SD, standard deviation; p, *p* value: *p*-values estimated with Mann-Whitney U test.

## Discussion

Vascular endothelial growth factor is a well-known promoter of angiogenesis and has been shown to be involved in the pathogenesis of wet AMD. Although many other methods have been explored, intravitreal anti-VEGF therapy is the only disease-modifying treatment for the retinal neovascular diseases (Plyukhova et al., 2020). The three anti-VEGF agents commonly used to treat retinal neovascular diseases include bevacizumab, ranibizumab, and aflibercept (Plyukhova et al., 2020). Intravitreal injections are simple to perform and the procedure for this treatment has been well established. However, intraocular injections are still considered to be invasive treatment (Avery et al., 2014), particularly for multiple injections. Some concern about intravitreal injection still exists, even though the injection is performed under sterile conditions (Grzybowski et al., 2018). In addition, the topic of inflammatory response has returned with the launch of a new anti-VEGF agent—brolucizumab (Yousef et al., 2020; Toro et al., 2021). For this drug, in phase 3 clinical trials and according to the FDA label, the incidence of ocular inflammation was higher (>4%) than other anti-VEGF agents (<1%) (Dugel, 2017; US Food and Drug Administration, 2019).

The most common treatment, in some countries, for ocular neovascular disease is bevacizumab, even though its use is off-label (Berg et al., 2015). Clinical observations have shown that the pharmacological effect of bevacizumab to handle retinal diseases is as safe and effective as to other anti-VEGF agents (Berg et al., 2015; Martin et al., 2020; Plyukhova et al., 2020). The CATT Research Group performed the largest bevacizumab-ranibizumab comparison study, which included 1,208 patients from 44 centers in the United States. Patients were put on a monthly or as-needed treatment scheme. Endophthalmitis was rare, occurring after 2 of 5,449 injections (0.04%) in 599 patients treated with ranibizumab injections (Martin et al., 2011). A similar incidence (0.07%, 4 of 5,508 injections) was observed in the 586 patients treated with bevacizumab (*p* = 0.49) (Reibaldi et al., 2019). However, one or more serious systemic adverse events occurred in 31.7% of ranibizumab-treated patients and in 39.9% of bevacizumab-treated patients (*p* = 0.004) (Martin et al., 2011).

Endophthalmitis is the most threatening complication associated with intravitreal injection (Reibaldi et al., 2019). However, clinical data suggests that most cases following injection were caused by medications contaminated during dose extraction (Merani and Hunyor, 2015).

Another safety concern surrounding intravitreal bevacizumab use, is ocular inflammation, that is known to occur after multiple intravitreal injections. Some reported cases associated with intravitreal injections suggest sterile endophthalmitis (Chong et al., 2010; Williams et al., 2016). These observations raise concerns of treating ophthalmologists regarding the risks and benefits of neovascular AMD treatments. Higher doses of intravitreal anti-VEGF agents have been shown to significantly increase the risk of intraocular inflammation (Rosenfeld et al., 2005). However, one study assessed anterior chamber inflammation after one intravitreal bevacizumab injection in eyes with exudative AMD and found no inflammatory response (Martin et al., 2012). Furthermore, a significant decrease from pre-injection values occurred in anterior chamber flare 7 days after injection. Our results, in accordance with Yeniad et al. (2011) did not show a decrease in ocular inflammation following injection. It may have been that Kiss et al. administered topical steroids following injection (Kiss et al., 2006), as it was commonly done in some centers. Unfortunately, this was not discussed by authors. In another study, a reduction in laser flare was observed two months after bevacizumab injections. However, only 8 patients were included in this analysis (Errera et al., 2014).

Even though we did not observe a decrease in ocular inflammation, we also did not observe an increase in inflammation. This finding is in agreement with pre-clinical *in vivo* studies that examined the ocular toxicity of four different intravitreal bevacizumab doses. A 5 mg dose of bevacizumab was not toxic to the retina, and only a few inflammatory cells in the vitreous were identified (Manzano et al., 2006; Xu et al., 2010).

The VEGF protein is known to provoke an inflammatory reaction by increasing vascular permeability and activating adhesion of leucocytes to the vascular endothelium (Adamis and Shima, 2005). It is also well-known that eyes with neovascular AMD have markedly increased VEGF levels and higher flare values than normal eyes (Kubota et al., 1994). Indeed, Kubota et al. have shown that flare values in eyes with age-related macular degeneration were 0.28 ± 0.18 mg/ml, being significant in comparison with the control (0.12 ± 0.05 mg/ml) (Kubota et al., 1994). Therefore, our control group was comprised of eyes with neovascular AMD to minimize baseline differences in ocular inflammation. The delayed therapy in this group of patients was related to insufficient health availability in our clinic and not to an unethical decision. Till November 2015 (our study was concluded in September 2015) treatment of patients with wet AMD has been an epidemiological and economical problem in Poland. Since that date special treatment program financed from public funds started and the situation slowly improved (Figurska et al., 2020). Mekjavic et al. have provided a comprehensive overview of the clinical and economic burden of wet-AMD and DME in Central and Eastern Europe and the status quo associated with their management (Jaki Mekjavic et al., 2019). Patients from our control group were fully aware of risks resulting from delayed treatment but, due to transportation barriers and reimbursement issues, decided to wait for injections in our hospital and be monitored for the time pending the therapy.

Prior studies have compared ocular inflammation in patients treated with intravitreal anti-VEGF for various exudative eye diseases (e.g., non-proliferative diabetic retinopathy, macular edema with branch or central retinal vein occlusion). No differences in anterior chamber inflammation were observed (Yeniad et al., 2011). Various anti-VEGF agents (bevacizumab, ranibizumab, and aflibercept) have also been compared. Blaha et al. found a small, but statistically significant, difference between the change in anterior chamber flare 1 day after intravitreal bevacizumab or intravitreal ranibizumab administration (Blaha et al., 2015). However, the small observed difference was not clinically relevant because no evidence of increased cell or are counts has been observed after routine use of these drugs (Blaha et al., 2015).

Recent reports of a new agent-brolucizumab and its possible side-effects have re-ignited interest in the cause of inflammatory reactions after intra-vitreous anti-VEGF drug administration. The mechanism of inflammation during anti-VEGF therapy remains unclear and is currently under investigation. Various theories suggest an immune response to the active molecule of the drug, other protein by-products within the drug or pH changes. One of the possible hypotheses for the pathogenic mechanism of this spectrum of events that is under investigation is the formation of local anti-bodies (Agrawal et al., 2013; Baumal et al., 2020; Haug et al., 2020). Clarification of the pathogenesis of inflammatory reactions after some anti-VEGF drugs is important also for clinical reasons. It is crucial to distinguish non-infectious from infectious intraocular inflammation, a severe vision-threatening condition that requires urgent evaluation and treatment.

## Conclusion

In conclusion, our results showed a good safety profile and lack of inflammatory response following multiple intravitreal bevacizumab injections. These observations confirm that multiple intravitreal bevacizumab administrations are safe with no risk for patients with exudative AMD, even though CATT study demonstrated that the proportion of patients with one or more systemic serious adverse events was higher with bevacizumab than ranibizumab. Further prospective studies with an adequate sample size calculation and longitudinal testing are mandatory to confirm our preliminary data.

## Data Availability

The raw data supporting the conclusion of this article will be made available by the authors, without undue reservation.
